# A144 ZENKER’S PERORAL ENDOSCOPIC MYOTOMY (Z-POEM) OUTCOMES IN TREATMENT OF ZENKER’S DIVERTICULUM: A RETROSPECTIVE CASE SERIES

**DOI:** 10.1093/jcag/gwad061.144

**Published:** 2024-02-14

**Authors:** M Youssef, C Ching Hui Yee, R Bechara

**Affiliations:** University of Toronto, Toronto, ON, Canada; Division of Internal Medicine, McGill University Faculty of Medicine and Health Sciences, Montreal, QC, Canada; Kingston Health Sciences Centre, Kingston, ON, Canada

## Abstract

**Background:**

Zenker’s diverticulum (ZD) is an outpouching of tissue in an area of the posterior hypopharynx called the Killian triangle. ZD is associated with dysphagia, regurgitation, cough and recurrent aspirations. Open surgical repair has been the traditional treatment approach but has high morbidity (0-40%) and mortality (0-9.5%). Endoscopic intervention with septotomy is a less invasive approach with lower complications and shorter recovery times. However, technical success rates range between 56.4-100%, and recurrences occur in 0-32% of patients. Recent studies have shown promising results for peroral endoscopic myotomy treatment of ZD (Z-POEM), although data is limited.

**Aims:**

In this first Canadian case series, we assess the safety and efficacy of Z-POEM, a novel approach for managing ZD.

**Methods:**

This is a retrospective case series of all patients who underwent Z-POEM for ZD at Kingston Health Sciences center between July 2021-2023. Data collected included patient demographics, symptoms [Kothari Haber Score (KHS)] pre and post Z-POEM, ZD size and prior treatments. Technical outcomes were also analyzed including the procedure time and the Z-POEM Difficulty Score (ZPDS), which ranges from 0-3 based on maneuverability, fibrosis, and oozing. Clinical success was defined as complete or near-complete resolution of symptoms (i.e. KHS ampersand:003C2), and recurrence was defined as KHS of 3 or more. Lastly, we recorded adverse events and hospital length of stay.

**Results:**

This case series included 9 patients (6 males and 3 females). The mean age ± standard deviation (SD) was 66.1 ± 10.64 years. One patient had two separate Z-POEM procedures and those were analyzed separately (total sample size = 10). The mean duration of symptoms before the procedure was 31.2 ± 10.12 months and the mean size of ZD was 2.25 ± 1.10 cm. Two patients had prior treatments before their Z-POEM procedure. The median preprocedural KHS was 6 (range 3-10) with dysphagia being the most commonly reported symptom in 80% of patients. In terms of technical outcomes, the mean procedural time was 32.3 ± 13.21 mins, and the median ZPDS score was 2. Most patients required 4-6 clips, with one patient requiring 11 clips for closure. No adverse events or periprocedural complications were reported. 9/10 patients (90%) were discharged one day after the procedure, and one patient required a longer stay (4 days) for observation. Clinical success was achieved in 8/10 patients (80%) of patients with a KHS score of 0 at mean 12 months follow-up. The remaining two patients (20%) had a KHS score of 3 at follow-up suggesting some persistent symptoms.

**Conclusions:**

Z-POEM is safe and effective in management of ZD with high technical and clinical success rates in the short-term. Larger-scale studies are required to determine the long-term clinical efficacy of Z-POEM in treatment of ZD.

**Funding Agencies:**

None

